# The Ubiquitous Premature Ventricular Complex

**DOI:** 10.7759/cureus.6585

**Published:** 2020-01-07

**Authors:** Cameron Koester, Abdisamad M Ibrahim, Michelle Cancel, Mohamed R Labedi

**Affiliations:** 1 Internal Medicine, Southern Illinois University School of Medicine, Springfield, USA; 2 Internal Medicine: Cardiology, Southern Illinois University School of Medicine, Springfield, USA

**Keywords:** pvc, premature ventricular contraction, premature ventricular complexes, cardiomyopathy, ablation, arrythmia, pvc-induced cardiomyopathy

## Abstract

Premature ventricular complexes (PVCs) are one of the most commonly encountered arrhythmias and are ubiquitous in clinical practice, both in the outpatient and inpatient settings. They are often discovered incidentally in asymptomatic patients, however, can cause myriad symptoms acutely and chronically. Long thought to be completely benign, PVCs have been historically disregarded without pursuing any further evaluation. Newer data have revealed that a high burden of PVCs with specific characteristics can significantly increase a patient's risk of developing PVC-induced cardiomyopathy. The aim of this literature review is to provide further clarification on the identification of high-risk PVCs, subsequent workup, and the currently available treatment options. PVCs arise from an ectopic focus within the ventricles. Patients with PVCs can be either asymptomatic or have severe disabling symptoms. The diagnostic workup for PVCs includes electrocardiogram (ECG) and 24-h Holter monitor to assess the QRS morphology and its frequency. A transthoracic echocardiogram (TTE) is done to look for structural heart disease and cardiomyopathy. Management of PVCs should be focused on identifying and treating the underlying causes, such as electrolyte abnormalities, substance use, and underlying structural heart disease. Beta-blockers are first-line therapy for symptomatic PVCs. Nondihydropyridine calcium channel blockers, classic antiarrhythmic agents, and amiodarone can be considered as second-line agents. Patients who are unable to tolerate medical therapy should undergo catheter ablation of the PVC focus to prevent PVC-induced cardiomyopathy. PVCs are common in clinical practice, and it is vital to identify patients at higher risk for PVC-induced cardiomyopathy to facilitate early intervention. Patients with no evidence of structural heart disease and infrequent PVCs should be monitored closely, while those who are symptomatic should be treated medically. For those who have failed medical therapy, catheter ablation of the PVCs focus is recommended. Catheter ablation has been shown to reduce PVCs burden and improve left ventricular ejection fraction (LVEF) in those with PVC-induced cardiomyopathy.

## Introduction and background

Ventricular premature beats (VPBs), premature ventricular complexes/contractions (PVCs), or ventricular extrasystole are ectopic beats that arise from within the ventricles. They are common and can occur in a wide variety of clinical scenarios and in a diverse population. Patients present either without symptoms, acutely symptomatic due to PVCs themselves, or with progressive symptoms from the cumulative effects that frequent PVCs can have on myocardial contractility. Historically, PVCs have been considered benign. However, PVC-induced cardiomyopathy has become more commonly recognized as a potential long-term consequence of PVCs. As PVCs are commonly encountered in clinical practice, it is imperative to understand the features that should prompt further evaluation. Early identification and intervention in patients with risk factors for PVC-induced cardiomyopathy can lead to drastically improved outcomes. While medications have had limited efficacy in reducing PVCs frequency and symptomatology, catheter ablation has become an increasingly utilized therapy. The aim of this literature review is to provide further clarification on the identification of high-risk PVCs, subsequent workup, and the currently available treatment options.

## Review

Electrocardiography

Premature ventricular complexes are defined electrocardiographically as a premature QRS complex with an abnormal morphology and duration greater than 120 ms [1]. It is classically followed by a broad T-wave with discordant deflection (opposite polarity) from the QRS complex. There are typically no preceding P waves. The impulse generated from the PVC can be propagated in a retrograde fashion to the atria and causes a compensatory pause following the PVC. This pause is due to atrioventricular (AV) nodal blockade as a result of depolarization from the retrograde impulse and subsequent refractory period of the AV node [1]. While the sinus node fires at an appropriate time, its impulse is not propagated, because the AV node has been rendered refractory. This causes an R-R interval that is exactly double the intrinsic R-R interval. 

The morphology of the QRS complex can be utilized to determine the origin of the ventricular premature beat. Left ventricular outflow tract (LVOT) and right ventricular outflow tract (RVOT) are the most common sites of origination of PVCs, and account for approximately two-thirds of all PVCs [2]. The ectopic origin can be localized in a similar fashion to identify the origin of ventricular tachycardia [3]. PVCs that originate from the RVOT have the morphology of a typical left bundle branch block (LBBB) and inferior axis on 12-lead electrocardiogram (ECG). Conversely, PVCs that originated from the LVOT have a right bundle branch block (RBBB) morphology with an inferior axis and precordial transition before V3. PVCs can originate from many other locations including the ventricular free wall, ventricular septum, aortic cusp, tricuspid/mitral annuli, pulmonary artery or papillary muscles, and all display unique QRS morphologies on 12-lead ECG [3]. 

Individuals with multiple abnormal QRS complexes are classified as having multifocal or polymorphic PVCs, which are associated with increased risk of mortality and nonfatal major cardiovascular events [4]. PVCs can also be defined by the pattern in which they occur relative to intrinsic beats. Bigeminy indicates a PVC following a single, normal QRS complex, whereas trigeminy indicates a PVC following two normal QRS complexes. Multiple successive PVCs are also given a distinct annotation. Two PVCs successively are a couplet, three PVCs successively are a triplet, and more than three successive PVCs are classified as ventricular tachycardia. Couplets can be further defined by the interval between the ventricular premature beat and the subsequent normal depolarization conducted from the sinoatrial (SA) node [1]. When the interval between the PVC and the normal beat is stable, it is termed fixed coupling. Variable coupling is when these intervals change from beat to beat. PVCs are considered "frequent" if they occur > 30 times per hour or > 20% of total heartbeats [5].

Prevalence and presentation

The prevalence of PVCs is variable depending on the duration of screening. In the general population, PVCs are identified in approximately 1%-4% of individuals when evaluating a single ECG [6-7]. When using a 24-h or 48-h Holter monitor for evaluation, the prevalence of asymptomatic PVCs is approximately 50% and 75%, respectively [8-9]. The Atherosclerosis Risk in Communities (ARIC) study found that approximately 6% of their participants had at least one PVC on a 2-min electrocardiogram [10]. Of these participants, PVCs were more frequent in patients with structural heart disease and hypertension. PVCs are more common in males than females, and the incidence increases with age, with a prevalence of approximately 70% in adults over the age of 75 years [2]. PVCs can be induced by myriad medications, substance use (tobacco, alcohol, cocaine, etc.) [1], electrolyte abnormalities (most commonly hypokalemia and hypomagnesemia), volume overload, and hypoxia. Though caffeine consumption has been thought to trigger PVCs, a study published in *The Journal of the American Heart Association* found that there was no relationship between chronic caffeine consumption and ventricular ectopy [11]. 

If PVCs are catecholamine-sensitive, exercise will increase the frequency of ectopy. One study found that PVCs that occur during an exercise stress test were associated with higher mortality risk [12]. A subsequent study revealed that PVCs occurring during the recovery phase of a stress test were a stronger predictor of death than those occurring during the exercise portion [13]. Sleep classically decreases the number of ectopic beats, however, in patients with sleep apnea or obesity hypoventilation syndrome, the number of PVCs is greatly increased during sleep [1]. In this scenario, PVCs are not induced by sleep, but rather the hypoxia caused by apneic and hypopneic episodes. 

Pathophysiology

The most likely underlying pathophysiology of PVCs involves three likely mechanisms: re-entry, triggered activity, and enhanced automaticity. Re-entry is thought to be the most common mechanism for both PVCs and ventricular tachycardia. This is an abnormal impulse that fails to propagate through the normal conduction system and instead travels in a retrograde fashion causing re-excitation and circular conduction. This is commonly seen in patients with previous myocardial infarction, scarring, or fibrosis with unidirectional conduction block. 

Triggered activity is defined by early or late after-depolarizations that may occur in the Purkinje fibers or in ventricular myocardium. They can be seen with hypokalemia, ischemia, infarction, cardiomyopathy, hypercalcemia, and drug toxicity (digoxin and QTc prolonging drugs). Early after-depolarizations are the result of reactivation of inward L-type calcium channels. Delayed after-depolarizations are thought to occur due to elevated intracellular calcium causing an increased inward current by the sodium-calcium exchange. This leads to an influx of sodium resulting in a triggered action potential [14]. 

Normal automaticity is the result of spontaneous phase four depolarization from the current in channels found in the sinus node [15]. Conversely, enhanced automaticity occurs when lower diastolic thresholds of transmembrane voltage, most commonly in the Purkinje fibers, results in premature depolarization and impulse propagation. This can be seen with electrolyte abnormalities, acute ischemia and in states of catecholamine excess. 

Evaluation

The PVCs usually do not cause any symptoms in asymptomatic individuals. They may be incidentally identified on physical exam or on routine screening ECG. Symptomatic patients either present due to the acute effects of PVCs (palpitations, chest pain, shortness of breath, lightheadedness, or dizziness) or due to the symptoms that arise from chronic PVCs and their cumulative effects on myocardial contractility. These are the symptoms that are typically seen in heart failure (dyspnea on exertion, orthopnea, and edema) and should be an indication for further investigation. In patients with pre-existing heart disease, frequent PVCs can cause dyspnea, angina, presyncope, syncope, and even hemodynamic compromise. 

 Initial workup includes a thorough history and physical exam, resting ECG, and ambulatory ECG monitoring for a minimum of 24 h. If patients are symptomatic and the initial 24-h monitoring is unrevealing, further monitoring should be considered using a 48-h monitor, an event recorder, or an implantable loop recorder. In most patients, especially those who are symptomatic, evaluation with a transthoracic echocardiogram (TTE) should be considered to evaluate for underlying structural abnormalities, as this will drastically change the management. Special attention should be paid to the ejection fraction (EF), left ventricular (LV) chamber size, valvular pathology, regional wall motion abnormalities, global hypokinesis, or more rare diagnoses such as arrhythmogenic right ventricular dysplasia or LV noncompaction cardiomyopathy. Further evaluation with exercise/chemical stress testing, nuclear myocardial perfusion imaging, coronary angiography, and cardiac MRI should also be considered on a case-by-case basis [16]. Cardiac MRI may be useful when making the diagnosis of arrhythmogenic right ventricular cardiomyopathy, infiltrative diseases such as cardiac amyloidosis, or for confirmation of LV noncompaction cardiomyopathy. 

It is also reasonable to evaluate for any underlying electrolyte abnormalities, with specific attention to serum potassium and magnesium levels [1]. Additional laboratory evaluation with urine drug screen, thyroid-stimulating hormone (TSH) level, cardiac biomarkers [troponin and brain natriuretic peptide (BNP)], and serum digoxin level should be considered when indicated [1]. Typically, an extensive evaluation is not warranted unless structural heart disease is identified. In this case, it becomes important to exclude other etiologies of cardiomyopathy, as PVC-induced cardiomyopathy remains a diagnosis of exclusion. It is often difficult to identify whether frequent premature ventricular contractions are the culprit or a manifestation of concomitant cardiomyopathy [17].

Prognosis

The prognostic significance of premature ventricular complexes is variable based on the patient’s cardiac history and the presence of any underlying cardiac dysfunction. In patients without underlying cardiac disease, PVCs have been historically considered benign with little clinical importance. However, several studies have demonstrated that PVCs have several potential long-term complications that addressed in multiple cardiology society guidelines [14, 16]. The most clinically important example is PVC-induced cardiomyopathy. The aforementioned ARIC study followed participants for more than 10 years and found that a single PVC on initial evaluation portended a greater than two-fold increase in mortality from both coronary artery disease and sudden cardiac death [18-19]. A separate study that evaluated the ECGs of 45,402 military veterans showed a significant increase in both all-cause and cardiovascular mortality in patients with PVC on routine ECG [20]. While these studies seem to demonstrate increased mortality from ventricular ectopy, there is little evidence to support prophylactic intervention to suppress PVCs in patients without underlying cardiac disease. The presence of PVCs should prompt closer evaluation for possible undiagnosed cardiac disease or reversible underlying etiology. In patients with known underlying ischemic or structural heart disease, PVCs also appear to have a negative prognostic effect. In the MADIT-CRT study, frequent PVCs showed a significantly higher risk of heart failure, ventricular tachycardia, ventricular fibrillation, and death [21].

In recent years there has been growing concern about the potential for patients with frequent ectopic beats to develop PVC-induced cardiomyopathy. Duffee et al. first introduced the concept of PVC-induced cardiomyopathy in 1998 with his publication in Mayo Clinic Proceedings titled, “Suppression of frequent premature ventricular contractions and improvement of LV function in patients with presumed idiopathic dilated cardiomyopathy” [22]. Since this paper, multiple studies have been published with the aim of further delineating which patients are at highest risk for developing this complication. Several factors have been identified that affect the likelihood of developing PVC-induced cardiomyopathy including the frequency or burden of PVCs, the origin of the PVC, the QRS duration, and PVCs that are asymptomatic [23]. 

The PVCs are often identified incidentally making it incredibly difficult to discern duration of exposure to PVCs, as a patient can be completely asymptomatic for long periods of time. Furthermore, these patients, especially those with very frequent PVCs, may be at higher risk for cardiomyopathy as the ectopy has likely gone unidentified for quite some time. This may be why patients with asymptomatic PVCs are at higher risk of developing cardiomyopathy. 

The burden or frequency PVCs have been shown to correlate with the degree of LV dilatation and systolic dysfunction [24-28]. According to the 2017 American Heart Association (AHA)/American College of Cardiology (ACC)/Heart Rhythm Society (HRS) guidelines on ventricular arrhythmias and the prevention of sudden cardiac death, frequent PVCs are defined as the presence of at least one PVC on a 12-lead EKG or > 30 PVCs per hour [14]. While this is a widely accepted guideline, several studies have utilized different criteria to define frequent PVCs. One study used a cutoff of > 20,000 PVCs in 24 h [24], others have used > 10,000 PVCs in 24 h [28] and greater than 20% of total beats [29-30]. More recent studies have shown that LV systolic dysfunction can be seen even in patients with a significantly lower PVCs burden [31]. An absolute cut-off point at which PVCs have a definitive adverse long-term outcome is yet to be definitively identified. However, one of the more frequently cited studies suggests that a PVC burden of >24% has a sensitivity of 79% and specificity of 78% in predicting which patients will go on to develop PVC-induced cardiomyopathy [29]. It has been speculated that a 24-h Holter monitor is not sufficient to fully quantify a patient's true PVC burden [32]. While PVC burden is an important risk factor, it is not the only variable at play as patients with infrequent PVCs (approximately 10% of all beats) can develop PVC-induced cardiomyopathy. Similarly, patients who have PVCs approaching 50% of all beats can have EFs that are well within normal limits [23]. It is important to quantify the PVC burden in order to have an understanding of the potential long-term consequences of the patient's PVCs, especially in patients with known LV systolic dysfunction.

It had been previously hypothesized that PVCs originating from the RVOT were the primary culprit for PVC-induced cardiomyopathy. This was thought to induce cardiac dyssynchrony in an analogous fashion to patients with an inherent LBBB or chronic right ventricular pacing [33-37]. When there are dyssynchronous contractions of the LV, the efficiency of LV systolic function is greatly reduced and asymmetrical hypertrophy can ensue. Both outcomes negatively impact myocardial kinetics and can lead to the development and progression of cardiomyopathy. Subsequent studies have shown that actually, PVCs originating from the epicardium are associated with the highest risk of cardiomyopathy. This is likely because PVCs with an epicardial origin have a significantly prolonged QRS complex, reflecting a greater degree of dyssynchronous depolarization of the LV [23]. This finding has led to the hypothesis that QRS duration also plays a key prognostic role in identifying patients at the highest risk for developing LV dysfunction from frequent PVCs. Multiple studies have shown that longer QRS duration (>140 or >150 ms) is an independent predictor of the development of cardiomyopathy [38-39].

Treatment

Treatment can be considered in patients who are symptomatic with a high burden of ectopic beats or frequent episodes of nonsustained ventricular tachycardia [16]. The first consideration in treating symptomatic PVCs is correcting the underlying cause. Specific attention should be paid to correcting electrolyte abnormalities, cessation of substance use, and management of comorbidities and underlying structural heart disease. 

Potassium and magnesium are the most important electrolytes to correct when treating PVCs. One study demonstrated that in patients hospitalized with acute myocardial infarction, a potassium concentration between 3.5 and 4.5 mmol/L was found to have the lowest mortality from ventricular arrhythmia [40]. In fact, this study showed that the incidence of ventricular arrhythmia did not increase unless the serum potassium was <3.0 or ≥ 5.0 mmol/L. Magnesium repletion should be provided in patients with concomitant hypokalemia and hypomagnesemia, to facilitate potassium repletion, and in patients with prolonged QTc intervals to prevent transformation into torsades de pointes. 

If treatment is indicated, beta-blockers are typically utilized as first-line therapy, as they generally have an excellent safety profile [41]. Catecholamine-sensitive or exercise-induced PVCs typically have an excellent response to the initiation of beta-blocker therapy. Additionally, PVCs in the setting of structural heart disease or myocardial infarction should be treated with beta-blockers as first-line therapy, as directed by guideline-directed medical therapy. Nondihydropyridine calcium channel blockers can be considered in patients with a contraindication for beta-blockers, as they have been found to successfully suppress PVCs originating from the outflow tract in patients with structurally normal hearts [42]. Antiarrhythmic therapy with class Ic agents (i.e. flecainide or propafenone) can be considered for patients who have a high PVC symptom burden and have not seen significant improvement after initiation of beta-blocker or calcium channel blocker therapy [43]. Unfortunately, these agents are proarrhythmic, increase mortality, and are thus contraindicated in patients with pre-existing coronary heart disease [44-45]. For patients with contraindications to class Ic antiarrhythmic therapy, amiodarone has proven to be effective in suppressing PVCs. However, due to the side effect profile, administration should generally be reserved for patients with underlying structural heart disease and significant symptoms [46]. Because of these serious side effects and medication interactions, amiodarone administration is more commonly used as a bridge to catheter ablation [14]. Sotalol has long been thought to suppress ventricular arrhythmias, however, it has not been shown to have mortality benefit [47]. In patients with systolic heart failure, sotalol was found to increase the risk of death [45]. 

Patients who cannot tolerate medical therapy should be referred to an electrophysiologist for consideration of ablation to prevent future cardiomyopathy [48], especially if the PVC burden is high [16]. The risk of cardiomyopathy is significantly higher in patients whose PVCs have a very wide QRS complex, arise from the epicardium, or occur greater than a quarter of all beats on 24-h Holter monitor [23, 29]. This cardiomyopathy is generally considered to be reversible, however, some degree of LV systolic dysfunction may persist after ablation of the ectopic focus. Patients who have already developed cardiomyopathy due to high-frequency PVCs should also be referred for radiofrequency ablation. Several studies, as well as a meta-analysis, have demonstrated that catheter ablation of PVCs originating from the RVOT significantly reduces ventricular ectopy burden and improves LVEF [25, 27, 29]. Ablation may also be indicated in patients with frequent PVCs that interfere with cardiac resynchronization therapy [23]. Ablation technology and mapping techniques have significantly improved over recent years, expanding the role of catheter ablation to higher risk epicardial and papillary PVCs [23].

Several cardiology societies have provided guidelines on the utilization of ablation for patients with PVCs. The 2017 AHA/ACC/HRS Guideline for Management of Patients with Ventricular Arrhythmia and the Prevention of Sudden Cardiac Death states that catheter ablation may be useful for patients with reduced LVEF suspected to be caused by frequent PVCs, defined as >15% of all beats, who have failed or could not tolerate antiarrhythmic medications [14]. The 2015 European Society of Cardiology Guideline recommends consideration of ablation therapy for patients with reduced LVEF and frequent symptomatic PVCs or nonsustained ventricular tachycardia [49]. Additionally, The European Heart Rhythm Association/Heart Failure Association recommends that patients with heart failure with reduced EF and a high burden of PVCs, defined as >10,000 PVCs/24 h, should be aggressively treated with catheter ablation if they have failed, declined, or are intolerant to antiarrhythmic therapy (especially if there is a single dominant PVC morphology) [50].

## Conclusions

Patients with PVCs should have a thorough evaluation for reversible causes, such as electrolyte abnormalities, substance use, and structural abnormalities. Patients should have a complete workup with an EKG, Holter monitor, and an echocardiogram to assess the QRS morphology, frequency of PVCs, and underlying structural heart disease. Patients with no evidence of structural heart disease and infrequent PVCs should be monitored closely, while those who are symptomatic should be treated medically. For those who have failed medical therapy, catheter ablation of the PVCs focus is recommended. Catheter ablation has been shown to reduce PVCs burden and improve LVEF in those with PVC-induced cardiomyopathy.
